# Self‐determination and Reciprocity: Ethical Research Engagement with Indigenous People Living with Dementia in Canada

**DOI:** 10.1002/alz70860_097728

**Published:** 2025-12-23

**Authors:** Pamela Roach, Jennifer D Walker

**Affiliations:** ^1^ University of Calgary, Calgary, AB, Canada; ^2^ McMaster University, Hamilton, ON, Canada

## Abstract

It is vital to incorporate thoughtful, rigorous and intentional approaches to the engagement of Indigenous people living with dementia and their families in research and policy. Engaging people living with dementia can be different when working in partnership with Indigenous communities. Authentic engagement requires engagement at multiple levels to preserve self‐determination across the entire research process.

The Indigenous research landscape in Canada is framed by both federal research requirements and community‐driven Indigenous ethical principles. The federal research requirements, articulated in the Tri‐Council Policy Statement: Ethical Conduct for Research Involving Humans, outline when and how community engagement should be undertaken with First Nations, Métis and Inuit individuals and communities. This includes when research will be done in partnership with Indigenous communities, or when research will be done with or analyses conducted with broader populations that may include and impact Indigenous people. These requirements provide a fail safe for academic Institutional Research Ethics Boards with which to hold researchers accountable to in a western model of research. It is perhaps more important, however, to consider community‐led Indigenous ethical principles when engaging Indigenous people living with dementia and their families in research. Concepts such as respect, reciprocity, relevance and responsibility guide all our work but there are also distinctions‐based approaches to ethical research relationships. Research with First Nations communities needs to consider the OCAP (Ownership, Control, Access, and Possession) research principles; research with Métis communities can refer to the Métis principles of research, and research with Inuit communities should implement Inuit research principles.

In this session, we present examples of our commitment to upholding and asserting Indigenous self‐determination while engaging Indigenous people with lived experience of dementia in research with the communities we each work with. Genuine relationships are the foundation and these relationships must be embedded and include people living with dementia in all stages of research, from initial research design, data collection, analysis and knowledge mobilization. We do this through sharing in ceremony, including traditional Knowledge Keepers and protocols and ensuring research and knowledge is co‐produced and owned by the communities leading the research.

